# A systematic review on the prevalence of, and risk factors for, eating disorders in systemic lupus erythematosus

**DOI:** 10.1177/13591053251338345

**Published:** 2025-07-12

**Authors:** Lauren Bruha, Fiona Duffy, Raahat Manrai, Helen Sharpe

**Affiliations:** 1 University of Edinburgh, UK; 2 NHS Lothian CAMHS, UK

**Keywords:** co-morbidity, disordered eating, eating disorders, SLE, systemic lupus

## Abstract

Systematic Lupus Erythematosus (SLE) is a chronic autoimmune disease with significant physical and psychological challenges. Case studies have highlighted examples of eating disorders (ED) developing in patients with SLE, but little is known about rates of co-morbidity or reasons for this. This systematic review investigated the prevalence of, and risk factors for, EDs in individuals with SLE. Exploring 21 studies with 3214 participants, most of which were cross-sectional with small sample sizes, findings indicated a similar prevalence of EDs in SLE populations as in the general population. However, individuals with SLE exhibited increased body image concerns, linked to higher disease activity, disease damage, and depressive symptoms. No studies explored risk factors for other ED symptoms (e.g. restriction, binge eating). This review emphasizes the need for further research to better understand the relationship between SLE and EDs, providing a basis for improved clinical practices and interventions for this population.

Lupus is a chronic, autoimmune disease in which the immune system attacks healthy tissues, affecting multiple organ systems throughout the body (Mayo Foundation for Medical Education and Research, 2023). Systemic Lupus Erythematosus (SLE), the most common form of the disease, affects roughly 3.4 million people worldwide (Centers for Disease Control and Prevention [CDC], 2022; Kiriakidou and Ching, 2020; Tian et al., 2022). SLE disproportionally affects females between the age of 15 and 45 years (Connelly and Morand, 2021; Conrad et al., 2023; LUPUS UK, 2023) and non-Caucasian ethnicities (Barber et al., 2021; CDC, 2022; Pons-Estel et al., 2010). There is no known specific aetiology of SLE, but a combination of environmental, genetic and hormonal factors have been implicated in onset (CDC, 2022; Mayo Foundation for Medical Education and Research, 2023).

Previous research has demonstrated a notable co-morbidity between autoimmune diseases and eating disorders (e.g. Hedman et al., 2019; Raevuori et al., 2014). In particular, high co-morbidity with eating disorders have been shown for Type I Diabetes (including inducing weight loss via insulin restriction), Crohn’s Disease, and Coeliac’s Disease (Falcão and Francisco, 2017; Fornari et al., 2001; Raevuori et al., 2014; Wotton et al., 2016; Zerwas et al., 2017). In terms of mechanisms, dietary restrictions necessary in some of these conditions may lead to high preoccupation with food, which can increase disordered eating behaviours. Furthermore, many medications used in the treatment of autoimmune diseases lead to changes in body composition, including weight gain, which can negatively impact body image (Colton et al., 2009, 2015; Quick et al., 2013). There may also be non-specific mechanisms, including the stress associated with living with a chronic condition (Raevuori et al., 2014).

Whilst SLE shares many characteristics with other autoimmune diseases, less research has focused on disordered eating in this population. Studies have demonstrated increased rates of other mental health problems, including anxiety, cognitive dysfunction and psychosis (Asano et al., 2013; Meszaros et al., 2012; Palagini et al., 2013) relative to the general population. Risk factors for mental health problems in people with SLE include genetic predisposition, environmental stressors, immune system dysfunction and the side effects of medications used to treat SLE (Asano et al., 2013; Meszaros et al., 2012; Palagini et al., 2013).

Given the significant psychological and physical challenges associations with SLE, such as chronic pain, persistent fatigue, anxiety and depression, as well as body composition changes associated with typically used medications, it is reasonable to hypothesize that similar mechanisms could contribute to the development of eating disorders in individuals with SLE. However, more research is required to fully understand this relationship in order to be able to make informed decisions about whether tailored prevention and treatment approaches may be needed in this group. Therefore, this systematic review had two aims: (1) to identify the prevalence of eating disorders among individuals with SLE; (2) to examine risk factors associated with eating disorders/disordered eating symptoms in SLE.

## Methods

This systematic review was reported using the Preferred Reporting Items for Systematic Review and Meta-Analyses (PRISMA) 2020 statement (Page et al., 2021). The protocol was registered on the International Prospective Register of Ongoing Systematic Reviews (PROSPERO) databases (CRD42023390024).

### Eligibility criteria

All observational studies (i.e. case reports, cross-sectional, cohort, or case-control studies) were included if they (1) measured a clinical diagnosis of an eating disorder, or eating disorder symptomology using a validated assessment tool; and (2) contained a sample with a diagnosis of SLE based on a formal diagnostic criteria or validated assessment tool. Standards for a SLE diagnosis included the International Classification of Disease (ICD), the European League Against Rheumatism and the American College of Rheumatology (EULAR/ACR; Aringer et al., 2019), and the Systemic Lupus Erythematosus International Collaborating Clinics (SLICC; Petri et al., 2012). If a study sample included individuals with various autoimmune diseases, the study was included if data from a subsample of SLE participants was available. Conference abstracts, reviews, qualitative studies and studies published prior to 1997 or in a language other than English were excluded.

### Information sources and search strategy

A comprehensive literature search of English articles was completed within the following databases: APA PsycINFO, Applied Social Science Index and Abstracts (ASSIA), Cumulative Index to Nursing and Allied Health Literature (CINAHL) Plus, EMBASE, PudMed (MEDLINE), Web of Science Core Collection, DART Europe, EBSCO Open Dissertations, Open Access Theses and Dissertations and ProQuest Dissertations & Theses Global. Each database was searched using keyword variants of lupus, autoimmune disease and eating disorder (Table 1). Two additional searches were completed on 26 April 2024 and 10 Jan 2025. LB hand search two journals (Lupus and Arthritis, and Rheumatology) and all reference lists of included articles were manually searched for additionally relevant studies. Authors of past conference abstracts and poster presentations related to the review were also contacted for any other relevant papers.

**Table 1.** Search strategy.

Line	Keyword Search
1	Lupus OR systemic lupus OR systemic lupus erythematosus OR SLE
2	Autoimmune disease OR autoimmune disorders
3	Eating disorder OR disordered eating OR feeding and eating disorder OR anorexia OR bulimia OR binge eating OR other feeding and eating disorder OR OSFED OR eating disorder not otherwise specified OR EDNOS OR disturbed eating behaviours OR eating pathology OR eating behaviours OR eating disorder symptomology OR disordered eating symptomology OR fasting OR dieting OR restrictive eating OR body image OR body image dissatisfaction OR body representation disturbances OR body mass OR body mass index OR BMI OR body composition OR quetelet index OR body weight OR purging OR vomiting OR laxatives OR diuretics OR extreme exercise
4	1 OR 2
5	3 AND 4

### Selection process

Following de-duplication, the titles and abstracts were screened by LB with 33% independently reviewed by RM. For all articles remaining, full texts were reviewed (100% LB, 50% RM). Minimal conflicts (8%, *k* = 7) occurred and all discrepancies were resolved through discussion between the reviewers.

### Data items

The following information was obtained from each study: authors, year of publication, country, study design, sample size, age, measurement tool of eating disorder outcome and SLE diagnoses. Relevant descriptive and inferential statistics were extracted. This included the number and percentage of participants diagnosed with an eating disorder within the SLE sample, and the association between any putative risk factors and eating disorder diagnosis or symptoms within SLE groups. Potential risk factors included gender, ethnicity, age of onset of SLE, Body Mass Index (BMI), disease activity, fatigue, pain, inflammation and medication use.

### Quality assessment

Joanna Briggs Institute (JBI) Critical Appraisal tools were used to rate the methodological quality across the different study designs (Aromataris et al., 2024). LB and RM independently evaluated the included studies, reporting similar appraisals. Minor discussions were completed (20%, *k* = 4) regarding whether certain items were not reported or were unclear in the studies.

### Synthesis method

A narrative synthesis was used to demonstrate the results of this systematic review. The narrative and data synthesis were reported using the Guidance on the Conduct of Narrative Synthesis in Systematic Review (Popay et al., 2006), as well as the Synthesis without Meta-Analysis (SWiM) reporting guidelines (Campbell et al., 2020). Tables were used to summarize the results of the included studies and collected data items.

### Certainty assessment

The certainty of evidence for each research question was assessed using the Grading of Recommendations Assessment, Development and Evaluation (GRADE) approach (Granholm et al., 2019).

## Results

**Figure 1.**
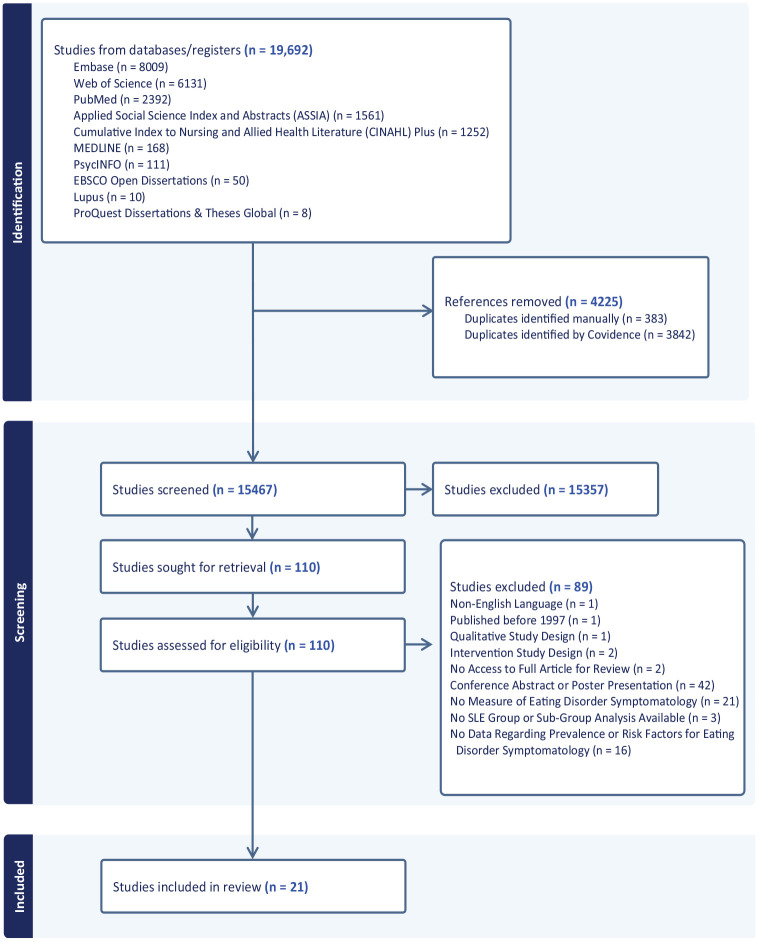
PRISMA flow chart of the included studies.

A total of 19,692 articles were retrieved, of which 4225 were duplicates. After screening the title and abstracts, and full texts, a total of 21 articles were included. Initially, 26 articles were identified; however, five assessed body image in SLE populations without a comparator for the prevalence or risk factors for EDs or disordered eating, resulting in a final sample of 21 studies. Figure 1 gives details of the study identification process. The included studies were published between 2005 and 2024. There were two case studies and one case series. Three studies examined the prevalence of eating disorders in those with SLE. Finally, 15 studies examined risk factors for eating disorder symptoms in SLE (10 cross sectional studies, 3 case control, 2 cohort studies). There was a total of 3214 study participants (3027 SLE; 187 controls), most of whom were female (85%).

### Risk of bias of included studies

Full risk of bias scoring can be found in Supplemental Materials Table S1. Among the case reports (*k* = 2) and case series (*k* = 1), there was a low to moderate risk of bias. Specifically, Toulany et al. (2014) had some risk of bias due to lack of clarity on consecutive recruitment and reporting of patient demographics. All of the prevalence studies (*k* = 3) were rated as low risk of bias. The cross-sectional studies (*k* = 10) demonstrated relatively low risk of bias, except in the following categories: identifying the strategies to deal with confounding variables and ensuring that the participants were free of the outcome before the study commenced. There was varied risk of bias in the case-control studies (*k* = 3). Jolly et al. (2011) and Seawell and Danoff-Burg (2005) were rated as having higher risk of bias as they did not control for confounding factors and did not match their controls to cases well. Finally, the cohort studies (*k* = 2) were rated as having a moderate to high risk of bias. Only 40% of the studies specifically acknowledged and stated their strategies to deal with confounding factors. Additionally, only one study addressed issues with incomplete follow-ups. However, all five studies measured the exposure and outcomes in a valid and reliable way.

### Findings from case reports and case series

The first case report (Hyla-Klekot et al., 2021) described a 16-year-old female who was diagnosed with Anorexia Nervosa (AN) in 2015; presenting with a BMI of 14.5 and exhibiting symptoms of hallucinations, joint pain and inflammation during her AN treatment. She was diagnosed with SLE in 2016 after continuing to experience joint and kidney issues. By 2017, her BMI improved to 18.9, demonstrated mild SLE activity, and no AN symptoms. The second case report (Wang et al., 2024) depicted a 14-year-old female who was diagnosed with SLE and 6 months later, Avoidant and Restrictive Food Intake Disorder (AFRID). The patient presented with a BMI of 11.9 and reported symptoms of substantial anxiety related to school, body weight and certain foods. She was hospitalized for severe malnutrition and SLE treatment. Minimal information was provided at the 3 month follow-up except BMI (15.3).

The case series (Toulany et al., 2014) included seven females with SLE and AN, six of whom were diagnosed with AN before or within 2 months of their SLE diagnoses, and while one was diagnosed with AN 15 months after receiving an SLE diagnosis. The average age of AN onset was 12.3 years with an average of 6.5 months passing between AN and SLE diagnoses. Five out of six no longer demonstrated AN symptoms after starting SLE treatment.

### Prevalence rates of eating disorders in people with SLE, and SLE in people with eating disorders

Only one study reported on the prevalence of later eating disorders in those with a SLE diagnosis (Hedman et al., 2019, Table 2). Of the 921 females diagnosed with SLE, 32 (3.5%) later received a diagnosis of any eating disorder (Hedman et al., 2019). For specific eating disorders, the rates were: 1.4% for AN, 0.8% for BN, and 3.0% for other eating disorders (i.e. BED or EDNOS). Comparisons to control participants showed that the prevalence of eating disorder were no higher in those with prior SLE to those without.

**Table 2.** Prevalence of eating disorders within SLE populations, and SLE within ED populations.

Study	Measurement of SLE	Measurement of ED	Prevalence Rate of ED within SLE	Comparison of Prevalence Rate of ED within SLE with Control Group	Prevalence Rate of SLE within ED	Comparison of prevalence rate of SLE within ED with control group
Hedman et al. (2019)	Health service records (ICD-10, Swedish ICD 8 & 9)	Health service records (DSM-IV, ICD-10, Swedish ICD 9)	Prevalence of ED after SLE Females Any ED: 32/921 (3.5%) AN: 13/921 (1.4%) BN: 7/921 (0.8%) OED: 28/921 (3.0%) Males Too few cases to calculate	Prevalence of ED after SLE Females^a^ Any ED: HR = 1.81 (1.12–2.91), *p* = 0.09 AN: HR = 2.39 (1.24–4.60), *p* = 0.06 BN: HR = 0.64 (0.16–2.55), *p* > 0.99 OED: HR = 1.83 (1.10–3.03), *p* = 0.12 Males Too few cases to calculate	Prevalence of SLE after ED Females Any ED: 32/24,386 (0.1%) AN: 13/11,372 (0.1%) BN: 7/5610 (0.1%) OED: 28/19,220 (0.1%) Males Too few cases to calculate	Prevalence of SLE after ED Females^b^ Any ED: HR = 1.33 (0.80–2.23), *p* = >0.99 AN: HR = 0.69 (0.26–1.85), *p* = >0.99 BN: HR = 2.01 (0.83–4.84), *p* = 0.76 OED: HR = 1.39 (0.62–3.11), *p* = 0.62 Males Too few cases to calculate
Raevuori et al. (2014)	Health service records (ICD-8, ICD-9, ICD-10)	Clinical assessment at admission to ED unit (DSM-IV, ICD-10 )	-	-	Lifetime prevalence of SLE Any ED: 6/2336 (0.3%) AN: 1/910 (0.11%) BN:2/1258 (0.16%) BED: 3/168 (1.75%)	Any ED: OR = 4.0 (1.29–12.4), *p* = 0.02^c^
Wotton et al. (2016)	Hospital admission records (inpatient; ICD-9, ICD-10)	Hospital admission records (inpatient; ICD-9, ICD-10)	Too few cases to calculate	Too few cases to calculate	Prevalence of SLE after ED Females AN: 7/8700 (0.08%) BN: 6/4783 (0.13%) Males Too few cases to calculate	Females^d^ AN: RR = 1.50 (0.60–3.11), *p* = 0.40 BN: RR = 2.66 (0.75–4.49), *p* = 0.13 Males Too few cases to calculate

AN: anorexia nervosa; BN: bulimia nervosa; BED: binge eating disorder; OED: other eating disorders; ICD: international classification of disease; DSM: diagnostic and statistical manual of mental disorders; HR: hazard ratio; RR: rate ratio; OR: odds ratio.

a compares likelihood of onset of ED in those with SLE compared to control participants without SLE.

b compares likelihood of onset of SLE in those with ED compared to control participants without ED.

c compares likelihood of SLE hospital admission in those with prior ED hospital admission compared to control participants without a prior ED hospital admission.

d compares lifetime prevalence of SLE in those with any ED compared with matched control participants without an ED.

Three studies estimated the prevalence rates of SLE in people with eating disorders (Table 2). Hedman et al. (2019) and Wotton et al. (2016) reported that 0.08%–0.10% of individuals with any eating disorder went on to develop SLE. These rates did not differ from the prevalence rates in control participants. Raevuori et al. (2014) reported a *lifetime* SLE prevalence of 0.3% in those with a hospital admission for any eating disorder. This was significantly higher (OR = 4.0) than the lifetime prevalence in matched control participants.

### Rates of ED symptomatology in SLE versus healthy controls

The only facet of ED symptomatology that was assessed in the case-control studies was body image. Three studies compared body image between individuals with SLE (*n* = 225) and healthy controls (*n* = 187), with mixed results (Supplemental Materials, Table S2). Ji et al. (2012) found that children with SLE reported poorer body image compared to healthy controls, with a very large effect (*d* = 2.30). In adult samples, Jolly et al. (2011) found significant poorer body image in adults with SLE compared with healthy controls (*d* = 0.72), whereas Seawell and Danoff-Burg (2005) found no differences (*d* = 0.24).

### Risk factors associated with body image difficulties in SLE

Fifteen studies assessed how putative risk factors were associated with body image difficulties in patients with SLE. Detailed study characteristics and specific findings of each study are shown in Table 3.

**Table 3.** SLE associations and risks of disordered eating symptomology.

Authors (Year) Country	Sample *(N, Age, Gender, and Ethnicity)*	Body image measure	Risk factors and measures	Results
Bourré-Tessier et al, 2014 Canada	*N* = 99 45.2 years (SD = 14.5) 97% female 74% Caucasian, 11% African American, 6% Asian, 9% Other ethnicities	LupusPRO - Body Image	Disease Activity (SELENA-SLEDAI)	Greater disease activity moderately associated with poorer body image (SMD = 0.55)
Chen et al. (2022)	*N* = 215 Average age undeterminable98% female Ethnicity not reported	BIDQ	Depression (HAD-S)	Higher rates of body image disturbances were weakly correlated with higher rates of depression (*r* = 0.39)
			Disease Activity (SLEDAI and MFI-20)	Greater disease activity strongly associated with poorer body image (*B* = 2.45). Higher rates of body image disturbances were moderately associated with increased fatigue (*r* = 0.40)
Devilliers et al. (2012) France	*N* = 185 39.6 years (SD = 10.5) 88% female 74% Caucasian, 9% Afro-Caribbean, 13% North African, 3% Asian	LupusQoL – Body Image	Disease Activity (SELENA – SLEDAI)	Greater disease activity weakly associated with poorer body image (SMD = 0.22) Greater disease activity (VAS/pain) weakly correlated with poorer body image (*r*_s_ = 0.38)
Faria et al. (2022) Portugal	*N* = 79 43.6 years (SD = 10.9) 98.7% female 96.2% Caucasian, 3.8% Other	LupusQoL – Body Image	Disease Activity (SLEDAI-2K)	Greater disease activity (pain, fatigue) weakly-moderately correlated with poorer body image (*r* = 0.394, *r* = 0.622) Greater disease activity weakly associated with poorer body image (SMD = 0.36).
			Disease Damage (SLICC/ACR-SDI)	Greater disease damage moderately correlated with poorer body image (SMD = 0.52)
Gaballah and El-Najjar (2019) Egypt	*N* = 94 36.9 years (SD = 14.1) 83% female Ethnicity not reported	LupusQoL – Body Image	Disease Activity (SLEDAI)	Greater disease activity moderately correlated with poorer body image (*r* = −0.41)
			Disease Damage (SLICC/ACR-SDI)	Greater disease damage strongly correlated with poorer body image (*r* = 0.79)
Gholizadeh et al. (2019) United States	*N* = 135 48.5 years (SD = 13.9) 92.6% female 45.2% Caucasian, 20.7% Hispanic, 14.1% African American/Black, 16.3% Asian/Pacific Islander, 3.7% Other/Mixed	LupusPRO – Body Image	Disease Activity (LupusPRO – Pain and Vitality)	Greater disease activity strongly associated with poorer body image (*B* = 0.627)
			Depression (HADS)	Greater depressive symptoms strongly correlated with poorer body image (*r* = −0.699)
Hosseini et al. (2014) Iran	*N* = 78 36.8 years (SD = 10.1) 100% female Ethnicity not reported	LupusQoL – Body Image	Disease Activity (SLEDAI-2K)	Body image moderately poorer in those with higher disease activity compared to lower disease activity (SMD = 0.61)
			Disease Damage (SLICC/ACR-SDI)	Body image moderately poorer in those with higher disease damage compared to lower disease damage (SMD = 0.62)
Ji et al. (2012) China	*N* = 164 (84 SLE; 80 Controls) SLE 15.3 years (SD = 1.4) 100% female Ethnicity not reported Control 15.7 years (SD = 1.5) 100% female Ethnicity not reported	SPPC – Physical Appearance	Depression (CDI)	Greater depressive symptoms moderately associated with greater appearance concerns (*r* = 0.758)
Jolly et al. (2011) United States	*N* = 165 (87 SLE; 78 Controls) SLE 42.4 years (SD = 13.1) 90% female Ethnicity not reported Control 38.7 years (SD 13.2) Gender not reported Ethnicity not reported	BIQLI	Age (Self Report)	Older age weakly associated with poorer body image (*r* = 0.21)
			BMI (Self Report)	BMI not associated with body image (*r* = 0.12)
			Depression (CES-D)	Greater depressive symptoms strongly associated with poorer body image (*r* = −0.54)
			Disease Activity (SELENA-SLEDAI)	Disease activity not associated with body image (*r* = 0.03)
			Disease Damage (SLICC/ACR-SDI)	Greater disease damage moderately correlated with poorer body image (*r* = −0.32)
Jolly et al. (2010) United States	*N* = 185 42.4 years (SD 12.8) 94% female 60% African American, 23% Caucasian, 12% Hispanic, 6% Asian	LupusQoL – Body Image	Age (Self-Report)	Older age was weakly associated with poorer body image (*r* = 0.15).
			Disease Activity (SELENA-SLEDAI)	Overall disease activity not related to body image (*r* = −0.05). For specific aspects of disease activity, only greater alopecia and greater leukopenia weakly correlated with poorer body image (*r* = −0.32, *r* = −0.29).
			Disease Damage (SLICC/AC-SDI)	Overall disease damage not related to body image (*r* = −0.15). For specific aspects of disease damage, only greater skin damage weakly correlated with poorer body image (*r* = −0.25)
			Gender (Self Report)	Body image marginally poorer in women compared with men (SMD = 0.21)
Jones and Kimble (2022) United States	*N* = 43 33.5 years (SD = 6.9) 100% female 100% African American	SIBID-S	Disease Activity (SF-36)	Higher disease activity (pain) was strongly associated with higher body image disturbance (*r* = 0.50)
			BMI (Self Report)	Higher BMI was moderately associated with higher body image disturbance (*r* = 0.38).
			Depression (PROMIS)	Higher depression symptoms moderately associated with higher body image disturbance (*r* = 0.38).
McElhone et al. (2010) United Kingdom	*N* = 322 45.0 years (SD = 13.4) 80% female 23% White, 4% Black, 3% Asian, 1% Chinese, 0.3% Mixed/Other, 69% Missing	LupusQoL – Body Image	Age (Self-Report)	Older age was weakly associated with poorer body image (*r* = 0.14)
			Disease Activity (BILAG)	Overall disease activity was weakly associated with poorer body image (*r* = −0.11) With musculoskeletal disease activity as moderately associated with poorer body image (*r* = − 0.20)
			Disease Damage (SLICC/ACR-DI)	Overall disease damage was weakly associated with poorer body image (*r* = −0.7)
Narupan et al. (2022) Canada	*N* = 185 42.2 years (SD = 10.8) 96% female Ethnicity not reported	BIS	Depression (PHQ-9)	Low body satisfaction strongly associated with increased odds of high depressive symptoms compared with high body satisfaction (OR = 18.34)
Seawell and Danoff-Burg (2005) United States	*N* = 83 (54 SLE; 29 Controls) SLE 47.4 years (SD not reported) 100% female 91% Caucasian, 6% African American, 3% Asian Control 44.7 years (SD not reported) 100% female 90% Caucasian, 5% African American, 5% Asian	MBSRQ – Appearance Evaluation	Depression (CES-D)	Higher depressive symptoms moderately associated with poorer body image (*r* = −0.65).
			Disease Activity (Visual Analogue (Pain) and Fatigue Severity Scale)	Greater disease activity (pain, fatigue) moderately associated with poorer body image (*r* = −0.35, *r* = −0.36).
Touma et al. (2011) Canada	*N* = 41 45.3 years (SD = 13.2) 90% female 59% Caucasian, 17% Black, 7% Asian, 17% Other	LupusQoL – Body Image	Disease Activity (SLEDAI-2K)	Body image was poorer in periods of disease flares compared to periods of improvement or remission of disease activity (unable to calculate SMD).

#### Disease activity

Thirteen studies examined the relationship between disease activity and body image disturbances in SLE participants. The majority of the studies found that higher disease activity was significantly associated with poorer body image, with the strength of associations ranging from weak to strong (Bourré-Tessier et al., 2014; Chen et al., 2022; Devilliers et al., (2012); Faria et al., 2022; Gaballah and El-Najjar, 2019; Gholizadeh et al., 2019; Hosseini et al., 2014; Jones and Kimble, 2022; McElhone et al., 2010; Seawell and Danoff-Burg, 2005; Touma et al., 2011). The only exception was Jolly et al. (2010, 2011), who found no association between disease activity overall and body image, but weak associations were found for specific symptoms including alopecia (Jolly et al., 2010). Other studies highlighted specific symptoms of pain (Jones and Kimble (2022) and musculoskeletal disease activity (McElhone et al. (2010), as being associated with poorer body image. All results are found in Table 3.

#### Disease damage

Six studies assessed the association between disease damage and body image. Five of these found greater disease damage in SLE patients was associated with poorer body image, with weak to strong effect sizes (Faria et al., 2022; Gaballah and El-Najjar, 2019; Hosseini et al., 2014; Jolly et al., 2011; McElhone et al., 2010; Table 3). The exception was Jolly et al. (2010), who reported no association between overall disease activity and body image, but a weak correlation between skin damage specifically and poorer body image.

#### Depression

Seven studies tested the association between depressive symptoms and body image in SLE patients, and all of these studies reported a significant association (Chen et al., 2022; Gholizadeh et al., 2019; Ji et al., 2012; Jolly et al., 2011; Jones and Kimble, 2022; Narupan et al., 2022; Seawell and Danoff-Burg, 2005; Table 3). Effect sizes ranged from moderate to strong. For example, Narupan et al. (2022) reported that low body satisfaction significantly increased the odds of patients with SLE having a high depression scores compared to those with high body satisfaction(Table 3).

#### Other risk factors

Additional factors explored in the included studies included age, BMI and gender (Table 3). Jolly et al. (2010, 2011) both found older age weakly correlated with poorer body image. Jolly et al. (2010) reported slightly poorer body image in women compared to men. Two studies assessed BMI and body image; Jolly et al. (2011) found no relationship, while Jones and Kimble (2022) reported a moderate association between increased BMI and higher body image disturbance.

### Certainty of evidence

The overall certainty of evidence for each aspect of this review were rated as very low to low, using the GRADE approach (Supplemental Materials, Table S3). These ratings were primarily based on the exclusively observational study designs, the small numbers of studies and low sample sizes and high indirectness in evidence as many studies were not directly assessing eating disorder symptomatology.

## Discussion

This systematic review aimed to explore the relationship between SLE and eating disorders, including the prevalence of eating disorders in those with SLE and risk factors for eating disorder symptoms in this population. Based on the limited evidence available, we found that the prevalence rates of eating disorders in people with SLE are similar to those in the general population. This is in contrast to findings in other autoimmune conditions, where rates of eating disorders seem to be elevated (Zerwas et al., 2017). While these findings demonstrate a co-occurrence of SLE and eating disorders in some individuals, there is no consistent indication that SLE itself increased the overall risk for these disorders (or vice versa). One study (Raevuori et al., 2014) did find a higher lifetime prevalence of SLE hospital admission amongst people admitted to hospital for an eating disorder (compared to those without an eating disorder). However, this study had very low cell counts (only 0.3% of participants with an eating disorder with a SLE admission) limiting the precision of the estimates.

This review did find some evidence of higher rates of body image concerns in individuals with SLE compared to healthy controls. This mirrors findings of elevated rates of body image concerns across other autoimmune conditions (Peters et al., 2021; Wabich et al., 2020). While body image concerns focused on weight and shape are a core diagnostic feature of anorexia nervosa and bulimia nervosa, current eating disorder assessment tools may not adequately capture the distinct and complex disordered eating behaviours in SLE. Body image distress impacted by physical manifestations of SLE (i.e. scarring, rashes, or changes in skin pigment) creates a further layer to self-perception, as individuals with SLE may engage in disordered eating behaviours to cope with visible symptoms rather than to solely alter weight. Hale et al. (2006) highlighted the importance of cosmetics to conceal skin damage in women in SLE, reporting the improvement in their quality of life. Because there is no cure for SLE, focusing on managing symptoms and addressing visible manifestations of SLE may help reduce body image concerns. Additionally, the development of assessment tool that measures SLE specific concerns may provide an accurate representation of disordered eating behaviours. This would allow for better interventions and improve quality of life.

Some risk factors for poor body image in those with SLE were specific to SLE populations, that is, higher disease activity and higher disease damage. Given the cyclical nature of SLE, individual often experience periods of flares and remissions. During disease flares, individuals experience an increase in symptoms such as joint pain, fatigue, hair loss (alopecia), and skin damage (Mayo Foundation for Medical Education and Research, 2023). Joint pain and fatigue can impact an individual’s ability to participate in physical activities, potentially leading to changes in body compositions that may increase body image concerns. Moreover, SLE flares are often managed with medications, such as corticosteroids, which have side effects like weight gain and hair loss. Studies from Hale et al. (2015) and Rodrigues et al. (2021) highlight how treatment side effects negatively impacted body image in individuals with SLE. It is notable that the review contained many studies with SLE populations with low to no disease activity, meaning the association between disease activity and body image may be underestimated here. Future work with participants with a range of levels of disease activity and damage would be valuable in understanding this association more clearly.

Other risk factors for body image in those with SLE, including age, BMI, gender and depression, are shared with the general population. These factors reflect currently identified risk factors for eating disorders and disordered eating (Barakat et al., 2023; Jacobi et al., 2004; Striegel-Moore and Bulik, 2007). This overlap of risk factors demonstrates that individuals with SLE experience body image concerns similar to the general population, but may be compounded by the complexity of SLE. The similarity in risk factors allows for existing interventions for EDs to be used to address body image concerns in individuals with SLE. Adjustments to prevention and treatment strategies may be required to account for specific aspects of SLE.

### Limitations of the review

This systematic review has several limitations which affect the level of confidence in the results. The majority of the studies were observational, which increased the risk of bias and limited the ability to draw temporal or causal conclusions. The estimates of effects were generally imprecise, given the small sample sizes of included studies. One major limitation is the heterogeneity of the included studies particularly with the study designs, populations and measurement tools. For example, the variety of measurement tools used to assess body image obstructs the ability to draw definitive conclusion, as some tools focused on body image in the context of quality of life while others were specifically designed to assess body image typically associated with disordered eating (i.e. weight and shape concerns). Moreover, the majority of studies lacked information regarding age, race, ethnicities, BMI, and disease severity in their samples, thus limiting the generalizability of the results to the broader SLE population. The lack of demographic characteristics makes it difficult to determine whether the results of this review are applicable to all individuals with SLE or only a subgroup. Capturing and including a wider range of demographics allows for a better understanding of how these factors may impact body image concerns, as well as prevention and treatment interventions.

### Implications for clinical practice and future research

Based on limited findings, this review does suggest that body image should be consideration in the care of those with SLE, particularly those with higher disease activity and more disease damage. Clinicians and practitioners need to be aware of the potential impact SLE has on an individual’s body image. The review gives very preliminary evidence to suggest that addressing specific symptoms, such as pain, fatigue and visible skin damage, may help reduce body image disturbances.

Future research should focus on exploring the shared and specific mechanisms driving the development of eating disorders and body image disturbances in SLE. Longitudinal studies could provide insights into how body image changes over time with disease progression and treatment, as well as the effectiveness of targeted interventions aimed at improving body image. Future work is needed to determine whether specific prevention programmes and/or treatment regimens are needed for those with SLE at risk of/with eating disorders.

### Conclusion

This systematic review provides important insights into the relationship between SLE, body image and eating disorders. Unlike in other autoimmune conditions, there does not seem to be an elevated prevalence of eating disorders in those with SLE. Elevated body image concerns in SLE are associated with higher disease activity and disease damage, as well as higher depression. The degree to which heightened body image concerns in this context increase the risk of developing future eating disorders remains unclear. Current studies are predominantly cross sectional, with small sample sizes, and do not specifically assess eating disorder symptomatology (e.g. restriction, binge eating). Future work is needed to explore how best to promote positive body image in those with SLE and to untangle any disease specific mechanisms that might underpin eating disorders in this group that could warrant more targeted intervention.

## Supplemental Material

sj-docx-1-hpq-10.1177_13591053251338345 – Supplemental material for A systematic review on the prevalence of, and risk factors for, eating disorders in systemic lupus erythematosus Supplemental material, sj-docx-1-hpq-10.1177_13591053251338345 for A systematic review on the prevalence of, and risk factors for, eating disorders in systemic lupus erythematosus by Lauren Bruha, Fiona Duffy, Raahat Manrai and Helen Sharpe in Journal of Health Psychology
