# Supplementary material for: A systematic review on the prevalence of, and risk factors for, eating disorders in systemic lupus erythematosus
Source: J Health Psychol. 2025 Jul 12;31(3):899–916. doi: 10.1177/13591053251338345 (PMC12949754; doi:10.1177/13591053251338345)
Supplement: sj-docx-1-hpq-10.1177_13591053251338345 – Supplemental material for A systematic review on the prevalence of, and risk factors for, eating disorders in systemic lupus erythematosus [file sj-docx-1-hpq-10.1177_13591053251338345.docx]

Supplementary Materials for *A systematic review on the prevalence of, and risk factors for, eating disorders in systemic lupus erythematosus* (Bruha, et al.)

**Table S1:** Critical Appraisal of Included Studies

| **Study Design** | **Numbered Item on the Critical Appraisal Tool** | | | | | | | | | | |
| --- | --- | --- | --- | --- | --- | --- | --- | --- | --- | --- | --- |
| Case Report | 1 | 2 | 3 | 4 | 5 | 6 | 7 | 8 |  |  |  |
| Hyla-Klekot et al. (2021) | Y | Y | Y | Y | Y | Y | N/A | Y |  |  |  |
| Wang et al. (2024) | Y | N | Y | N | N | N | N/A | Y |  |  |  |
|  | 100% | 50% | 100% | 50% | 50% | 50% | 0% | 100% |  |  |  |
| Case Series | 1 | 2 | 3 | 4 | 5 | 6 | 7 | 8 | 9 | 10 |  |
| Toulany et al. (2014) | Y | Y | Y | Y | N | N | Y | Y | Y | N/A |  |
|  | 100% | 100% | 100% | 100% | 0% | 0% | 100% | 100% | 100% | 0% |  |
| Case-Control | 1 | 2 | 3 | 4 | 5 | 6 | 7 | 8 | 9 | 10 |  |
| Ji et al. (2012) | U | Y | N | Y | Y | Y | N | Y | N/A | Y |  |
| Jolly et al. (2011) | N | Y | Y | N | Y | Y | U | Y | N/A | U |  |
| Seawell and Danoff-Burg (2005) | Y | Y | N | Y | N | Y | U | N | N/A | Y |  |
|  | 33% | 100% | 33% | 67% | 67% | 100% | 0% | 67% | 0% | 67% |  |
| Cohort | 1 | 2 | 3 | 4 | 5 | 6 | 7 | 8 | 9 | 10 | 11 |
| Devilliers et al. (2012) | Y | N/A | Y | U | N | Y | Y | Y | U | Y | Y |
| Touma et al. (2011) | Y | N/A | Y | U | N | N | Y | Y | U | U | N/A |
|  | 80% | 60% | 100% | 40% | 40% | 80% | 100% | 80% | 0% | 20% | 80% |
| Cross-Sectional | 1 | 2 | 3 | 4 | 5 | 6 | 7 | 8 |  |  |  |
| Bourre-Tessier et al. (2014) | Y | Y | Y | Y | U | N | Y | Y |  |  |  |
| Chen et al. (2022) | Y | Y | Y | Y | Y | Y | Y | Y |  |  |  |
| Faria et al. (2022) | Y | Y | Y | Y | Y | Y | Y | Y |  |  |  |
| Gaballah and El-Najjar (2019) | Y | Y | Y | Y | U | N | Y | Y |  |  |  |
| Gholizadeh et al. (2019) | Y | Y | Y | Y | N | N | Y | Y |  |  |  |
| Hosseini et al. (2014) | Y | Y | Y | Y | N | N | Y | Y |  |  |  |
| Jolly et al. (2010) | Y | Y | Y | Y | Y | N | Y | Y |  |  |  |
| Jones and Kimble (2022) | Y | Y | Y | U | U | N | Y | Y |  |  |  |
| McElhone et al. (2010) | Y | Y | Y | N | U | Y | Y | Y |  |  |  |
| Narupan et al. (2022) | Y | Y | Y | Y | Y | U | Y | Y |  |  |  |
|  | 100% | 100% | 100% | 80% | 40% | 30% | 100% | 100% |  |  |  |
| Prevalence | 1 | 2 | 3 | 4 | 5 | 6 | 7 | 8 | 9 |  |  |
| Hedman et al. (2019) | Y | Y | Y | N | Y | Y | Y | Y | N/A |  |  |
| Wotton et al. (2016) | Y | Y | Y | N | Y | Y | Y | Y | N/A |  |  |
| Raevuori et al. (2014) | Y | Y | Y | N | Y | Y | Y | Y | N/A |  |  |
|  | 100% | 100% | 100% | 0% | 100% | 100% | 100% | 100% | 0% |  |  |

**Table S2**. Body Image in Healthy Controls vs Systemic Lupus Erythematosus

| **Study** | **SLE Participants**  **n** | **Controls**  **n** | **Measurement of Body Image** | **Results**  **M** ± SD, t-test | **Standardized Mean Difference** |
| --- | --- | --- | --- | --- | --- |
| Ji et al. (2012) | 84 | 80 | Self-Perception Profile for Children - Physical Appearance Domain | SLE: 13 ± 2.8  Controls: 20 ± 3.1 t = 15.2, p < 0.05 | 2.30 |
| Jolly et al. (2011) | 87 | 78 | Body Image Quality of Life Inventory | SLE: 0.8 ± 1.3 Controls: 1.7± 1.2 t = 4.6, p < 0.001 | 0.72 |
| Seawell and Danoff-Burg (2005) | 54 | 29 | Multidimensional Body Self-Relation Questionnaire - Appearance Scales | SLE: 3.02 ± 0.77  Controls: 3.22 ± 0.89 t = 1.07, p = 0.20 | 0.24 |

**Table S3:** Certainty of Evidence, GRADE Assessment

| **Research Question** | **GRADE** | **Justification** |
| --- | --- | --- |
| *Prevalence Rates* | | |
| Eating Disorder Prevalence Within SLE Sample | Low | All though there were no concerns relating to risks of bias, inconsistency, indirectness, imprecision, or publication bias, due to the observational study design, the GRADE remains low as no factors allowed for the increase in quality of evidence status. |
| SLE Prevalence Within Eating Disorder Sample | Very Low | A high risk of bias was identified due to the failure to adequately identify confounding factors and complete follow ups. |
| *Associations and Risk Factors* | | |
| *Controlled Studies* | | |
| Healthy Controls vs SLE | Very Low | The high variability in effect sizes provided inconsistency across the study results and a risk of bias due to the inability to accurately match cases to controls. |
| *Uncontrolled Studies* | | |
| Disease Activity | Very Low | A high risk of bias was identified due to the study design variation. Additionally, the lack of generalizability to the wider SLE population and indirectness to answer the research question impacted the overall GRADE. |
| Disease Damage | Very Low | A high risk of bias was identified due to the study design variation. Additionally, the lack of generalizability to the wider SLE population and indirectness to answer the research question impacted the overall GRADE. |
| Depression | Very Low | A high risk of bias was identified due to the study design variation, and the difference in populations provides an indirectness of evidence. |
| Age | Very Low | A high risk of bias was identified due to the study design variation. |
| BMI | Very Low | A high risk of bias was identified due to the study design variation. An inconsistency of results also impacted the GRADE level. |
| Gender | Very Low | A lack of generalizability to the wider SLE population and indirectness to answer the research question impacted the overall GRADE. |
| *Footnotes* | | |
| Per the GRADE handbook, all observational studies start at a low level quality of evidence unless strengths allow for the level to be increased. | | |
